# Imaging Wheat Canopy Through Stereo Vision: Overcoming the Challenges of the Laboratory to Field Transition for Morphological Features Extraction

**DOI:** 10.3389/fpls.2020.00096

**Published:** 2020-02-18

**Authors:** Sébastien Dandrifosse, Arnaud Bouvry, Vincent Leemans, Benjamin Dumont, Benoît Mercatoris

**Affiliations:** ^1^Biosystems Dynamics and Exchanges, TERRA Teaching and Research Centre, Gembloux Agro-Bio Tech, University of Liège, Gembloux, Belgium; ^2^Plant Sciences, TERRA Teaching and Research Centre, Gembloux Agro-Bio Tech, University of Liège, Gembloux, Belgium

**Keywords:** wheat, stereo vision, crop phenotyping, canopy height, leaf area index, mean tilt angle, leaf angle distribution

## Abstract

Stereo vision is a 3D imaging method that allows quick measurement of plant architecture. Historically, the method has mainly been developed in controlled conditions. This study identified several challenges to adapt the method to natural field conditions and propose solutions. The plant traits studied were leaf area, mean leaf angle, leaf angle distribution, and canopy height. The experiment took place in a winter wheat, *Triticum aestivum* L., field dedicated to fertilization trials at Gembloux (Belgium). Images were acquired thanks to two nadir cameras. A machine learning algorithm using RGB and HSV color spaces is proposed to perform soil-plant segmentation robust to light conditions. The matching between images of the two cameras and the leaf area computation was improved if the number of pixels in the image of a scene was binned from 2560 × 2048 to 1280 × 1024 pixels, for a distance of 1 m between the cameras and the canopy. Height descriptors such as median or 95th percentile of plant heights were useful to precisely compare the development of different canopies. Mean spike top height was measured with an accuracy of 97.1 %. The measurement of leaf area was affected by overlaps between leaves so that a calibration curve was necessary. The leaf area estimation presented a root mean square error (RMSE) of 0.37. The impact of wind on the variability of leaf area measurement was inferior to 3% except at the stem elongation stage. Mean leaf angles ranging from 53° to 62° were computed for the whole growing season. For each acquisition date during the vegetative stages, the variability of mean angle measurement was inferior to 1.5% which underpins that the method is precise.

## Introduction

To overcome the double challenge to increase crop yield while limiting inputs, the development of high-throughput non-destructive phenotyping methods has emerged as a hot research topic. Many advancements have been made for indoor high-throughput set-ups (Perez-Sanz et al., 2017), whereas natural conditions such as wind or the variability of sunlight pose challenges for outdoor image acquisition and related treatment. In the field, the extraction of plant traits from a canopy structure also remains a complex task due to organ overlapping, especially for dense crops such as cereals. The development of robust methods to automatically measure morphological plant traits in field conditions is still required (Gibbs et al., 2017).

This paper focuses on the measurement of four morphological traits of great agronomic interest. (i) Leaf Area Index (LAI), which is the area of one side of leaves above one square meter of ground, expresses the photosynthetically active area. This parameter is also relevant to scale up the gas exchanges from leaf to canopy level (Bréda, 2003). As an indicator of crop development, it can help to manage nitrogen inputs. (ii) Mean tilt angle (MTA) is the average angle between the leaf segments and the horizontal ground. (iii) Leaf angle distribution (LAD) is the statistical distribution of leaf face angles. LAD and MTA condition light interception. The knowledge of LAD is useful for some methods aiming at estimating LAI based on gap fraction, which is the fraction of soil observed in a viewing direction, determined thanks to segmented 2D images or transmittance measurements (Weiss et al., 2004). In addition, LAD is a key trait to identify wheat varieties (Yanli et al., 2007). (iv) Finally, canopy height is an indicator of the risk of lodging and can be a criterion to discriminate weeds and crops (Piron et al., 2009). Moreover, height can provide information on yield because stressed plants can be shorter (Constantino et al., 2015).

The simultaneous and direct measurement of those morphological traits is conceivable with 3D proximal sensing techniques. Commonly used 3D acquisition devices are Light Detection And Ranging (LiDAR), time of flight cameras, mono and multi-view stereo vision, and structure from motion. LiDAR sensors scan the scene with lasers to obtain a 3D point cloud. This technique is widely used and provides precise and dense canopy models but the sensors are expensive (Li et al., 2014) and a combination with a RGB camera is required to obtain accurate color information, although some LiDAR devices provide intensity of the signal that help identifying green parts. Such a measurement takes more time than passive measurement and it is moreover necessary to increase the scanning time to increase the spatial resolution (Gibbs et al., 2017). As a result, this technique remains an issue in field conditions due to the wind-induced motion of leaves. Time of flight cameras illuminate the scene and compute depths for each pixel according to the time taken by the light to reach the objects. As the whole scene is illuminated simultaneously, time of flight cameras solve the scanning time problem. They are suitable for indoor measurements but the need of active light diminishes the performances of image acquisition under strong sunlight (Kazmi et al., 2012; Perez-Sanz et al., 2017). Binocular stereo vision relies on two cameras to compute depth by triangulation. The system is low-cost, simple, compact, allows quick acquisition and can operate in natural sunlight conditions. Its main drawbacks are the errors in depth measurement related to poor stereo matching, the computational requirements for the stereo matching algorithms and the influence of overlapping leaves. Multi-view stereo systems help to improve the quality of the depth map and the management of overlapping parts of the canopy. Using multiple cameras arranged around the scene of interest is suitable for indoor environment as realized by Scharr et al. (2017) and Hui et al. (2018) but is more challenging to implement in the field. Finally, structure from motion relies on the displacement of a single camera to reconstruct the scene. Jay et al. (2014) have efficiently implemented such system in field conditions to retrieve crop height and area. Its main drawback compared to stereo vision is the bigger amount of data to store and process.

As a result, stereo vision appears as a simple and robust way to study canopy architecture in field conditions. High-throughput plant phenotyping approaches using stereo vision have been developed in laboratory by He et al. (2003); Andersen et al. (2005); Biskup et al. (2007); Lin et al. (2011), and Tilneac et al. (2012). Only few in-field approaches have been proposed. Kise and Zhang (2008) used a stereo system for crop rows detection. Ivanov et al. (1994) applied stereo vision to study the leaf angle and area in a maize canopy. Müller-Linow et al. (2015) have tested stereo-imaging on sugar beet in natural conditions. For cereals, the task is more challenging because of homogeneous leaves texture and complex canopy architecture made of thin and long leaves. Leemans et al. (2013) introduced a method for area and angle computation for winter wheat.

This study aims at developing a proximal stereo vision system to measure LAI, MTA, LAD, and canopy height of winter wheat in field conditions. The first goal is to analyze the challenges encountered to adapt the stereoscopic method from single-pot in indoor controlled conditions to complex natural canopy and to propose solutions to these challenges. The second goal is to compare image-based measurements with manual conventional measurements and quantify the errors.

## Materials and Methods

### Field Experiment and Data Collection

The experiment took place in a field dedicated to agronomic trials during the 2018 season, located in Lonzée, Belgium (50° 32' 58' N and 4° 44' 08'' E). The experiment concerned 64 micro-plots of 1.8 × 6 m planted with winter wheat (*Triticum aestivum* L. “Edgar”), sowed with a density of 250 grains/m² on October 13, 2017. The row spacing was 0.14 m. The micro-plots were fertilized three times (at tillering, stem elongation and flag leaf stages) with 27 % ammonium nitrate. That nitrogen fertilization was applied following 11 modalities combining inputs of 0, 30, 60, and 90 kg of nitrogen per hectare in four replicates (see **Supplementary Material** for thorough information on field trial).

Manual reference measurements were performed to calibrate and validate vision methods. To measure LAI, leaves were collected on 0.5 m of a row in 20 of the 64 plots, laminated with transparent adhesive cover on paper sheets and scanned. May 24, 2018, when the spikes were not out yet, heights were manually measured at the insertion of flag leaf for 36 tillers per micro-plot (the plots were systematically divided into 12 zones in which three tillers were randomly selected). Insertion of flag leaf was chosen to perform repeatable height measurements. Such measurements on the tiller have the advantage to be independent from leaf orientation and does not necessitate to stretch leaves. The reason of this measurement before spike heading was to assess the ability of manual measurements to record plant height at a vegetative stage, although wheat height is conventionally measured on spikes (Pask et al., 2012). June 05, heights were manually measured at spike tops for 36 spikes per micro-plot. No reference measurements for MTA and LAD were performed in the field because of the curved shape of leaves. Average wind speed measurements were recorded by a sonic anemometer from the Lonzée ICOS station (50° 33' 06'' N and 4° 44' 46'' E) located in a neighboring plot.

Images were acquired in the field at the following dates: April 09, April 11, April 23, April 30, May 02, May 16, May 24, May 30, and June 05, 2018 under various light conditions with no artificial shadowing, so that the robustness of imaging methods to natural light could be tested. At each date, four pairs of images were taken per micro-plot. The image acquisition platform was designed to capture nadir frames of the wheat canopy at a distance of about 1 m. The two cameras used to form the stereo vision device were GO-5000C-USB from JAI group equipped with a 2560 × 2048 CMOS sensor and a RGB Bayer filter. The objectives were Kowa LM16HC with a focal length of 16 mm. The iris aperture was set to F2.8 and the focus to 1 m. The baseline (distance between the centers of the two camera sensors) was 50 mm and optical axes were parallel. The height of the stereo vision device was adjusted at each acquisition date to keep a distance of approximately 1 m between the canopy and the sensors. At this distance, the footprint of the images was around 0.5 m². The baseline and the camera height were calibrated to acquire images with an appropriate spatial resolution combined with large scene to account for intra-plot variability. The stereo vision device was calibrated using a 9 × 6 checkerboard (square side of 40 mm) and Matlab Stereo Camera Calibrator App according to the method proposed by Zhang (2000). Parameters obtained by calibration are rotation and translation matrix between the two cameras, focal lengths, and distortion coefficients. The calibration error was 0.32 pixels. Images were stored with a color resolution of 12 bits per pixel to take full advantage of the hardware. Additionally to the field measurements, images of leaves of known area and inclination were captured in laboratory to investigate the measurement errors. The target was made of three leaves stuck on a flat wooden board. To test the area computation, 20 positions of the target were captured. The positions were generated by combining rotations of the board along the three perpendicular directions of a 3D space from less than 75° in each direction, relative to a plane perpendicular to the optical axis of the cameras. To test the angle measurement, ten positions of the target were captured. Those positions were generated by tilting the target from 0 to 75° in one direction.

### Depth Mapping by Stereo Vision

**Figure 1:**
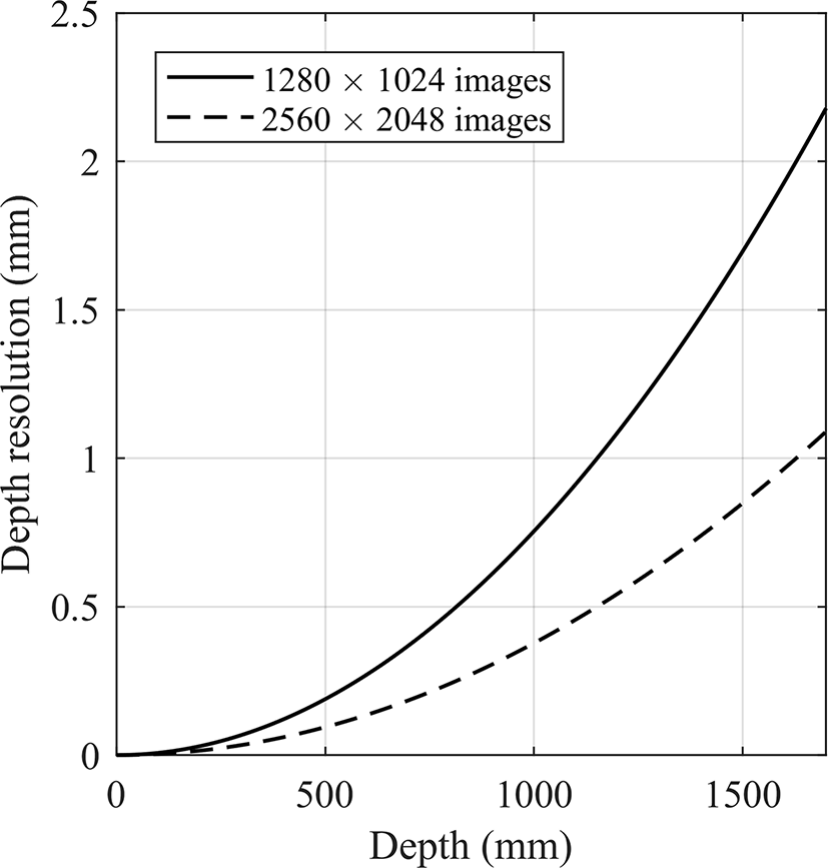
Depth resolution versus depth for two image sizes used in this study.

Exploiting the overlaps between left and right images to compute depth required several steps. Firstly, the rectification process consisted in aligning images so that a same point of the scene appeared at the same y-coordinate in the two images. This was performed by Bouguet's algorithm thanks to the calibration parameters of the system (Bradski and Kaehler, 2008). This rectification algorithm also relies on calibration parameters to account for radial lens distortion. The rectified images were converted to grayscale. In order to reduce the effect of noise on ulterior 3D computations, the grayscale image size was reduced to 1280 × 1024 pixels by averaging the pixel values on each 2 × 2 square. The second main step was the stereo matching which consisted in finding corresponding pixels in right and left images. The difference of x-coordinate of corresponding pixels gave the disparity between pixels. Stereo matching was performed with the Semi-Global Block Matching algorithm (SGBM) proposed by Hirschm (2007). The principle is to detect corresponding pixels by means of similar neighborhoods. The two most important parameters are the matching window size, which is the size of a side of investigated neighborhoods, and the disparity range, which corresponds to the maximal possible disparity. Matching window sizes of 5, 9, 15, and 19 pixels were tested. A window size of 15 is the default value, while 5 is the minimum value. The two other values were chosen to test other configurations, one between the default and the minimum value (9) and the other greater than the default value (19). Disparity range was automatically adjusted for each image pair if disparities peaked at the maximum allowed value. The disparity estimation was also controlled by post filtering based on minimum uniqueness value, set to 5, to remove false matches (Bradski and Kaehler, 2008). The complex texture of images acquired in natural conditions resulted in incomplete disparity maps which were filled by the method proposed by Yun (2012). This method performs interpolation only if reliable information is available in the neighborhood. The last step consists in computing depths, which are inversely proportional to disparities. For each pixel in the left frame of a pair of stereo images, considered as the reference, the depth to the camera is given by

$$Z=\frac{b\;f}{d}$$

where *b* is the baseline (m), *f* is the focal length (pixel), *d* is the disparity (pixel), and *Z* is the distance (m) between the observed object and the camera, commonly referred to as depth. The result of this whole step of image processing is a depth map, showing distances between objects in the scene and the cameras. As presented in **Figure 1**, depth resolution depends on depth.

### Image Segmentation by Color Processing

All image treatments were realized with Matlab R2016a. Images acquired before spike emergence stage, May 24, were separated into two classes: soil and leaves. The segmentation method was based on a support vector machine (SVM) classifier trained with the components of RGB and HSV color spaces. According to Hamuda et al. (2017), the addition of the HSV color space helps to obtain a segmentation more robust to natural light conditions. The use of machine learning helps to deal with complex situations containing enlightened and shadowed canopy elements. To train and evaluate the classifier, 10000 pixels were selected in a set of images representative of the different acquisition dates and conditions. The selected pixels were split so that 70% were dedicated to training and 30 % to validation. The last step of the process consisted in median filtering with a window of 5 × 5 pixels to remove segmentation noise on the resulting binary image.

Images acquired at flowering stage, June 05, contains spikes and were segmented into three classes: soil, leaves, and spikes. SVM providing binary outputs, three classifiers were combined according to the “Error Correcting Output Codes” principle (Dietterich and Bakiri, 1994). Moreover, color information is not sufficient to distinguish spikes at their early development stages because they are as green as leaves. For this reason, in addition of RGB and HSV components, height and texture predictors have also been used to train the SVM. Texture predictors of a pixel are (i) the average of pixels intensities over a 7 × 7 square centered on the considered pixel and (ii) the average of the squared differences of intensities between each pixel and the central pixel of the neighborhood. These parameters aim at taking into account the differences between the grainy texture of spikes and the smooth texture of leaves. To be independent of the camera-ground distance, the considered height predictor for each pixel was the difference between the 95th percentile of heights and the height of this pixel. To train and evaluate the classifier, 5000 pixels were selected with 70 % dedicated to training and 30 % to validation.

### Canopy Height Estimation

**Figure 2:**
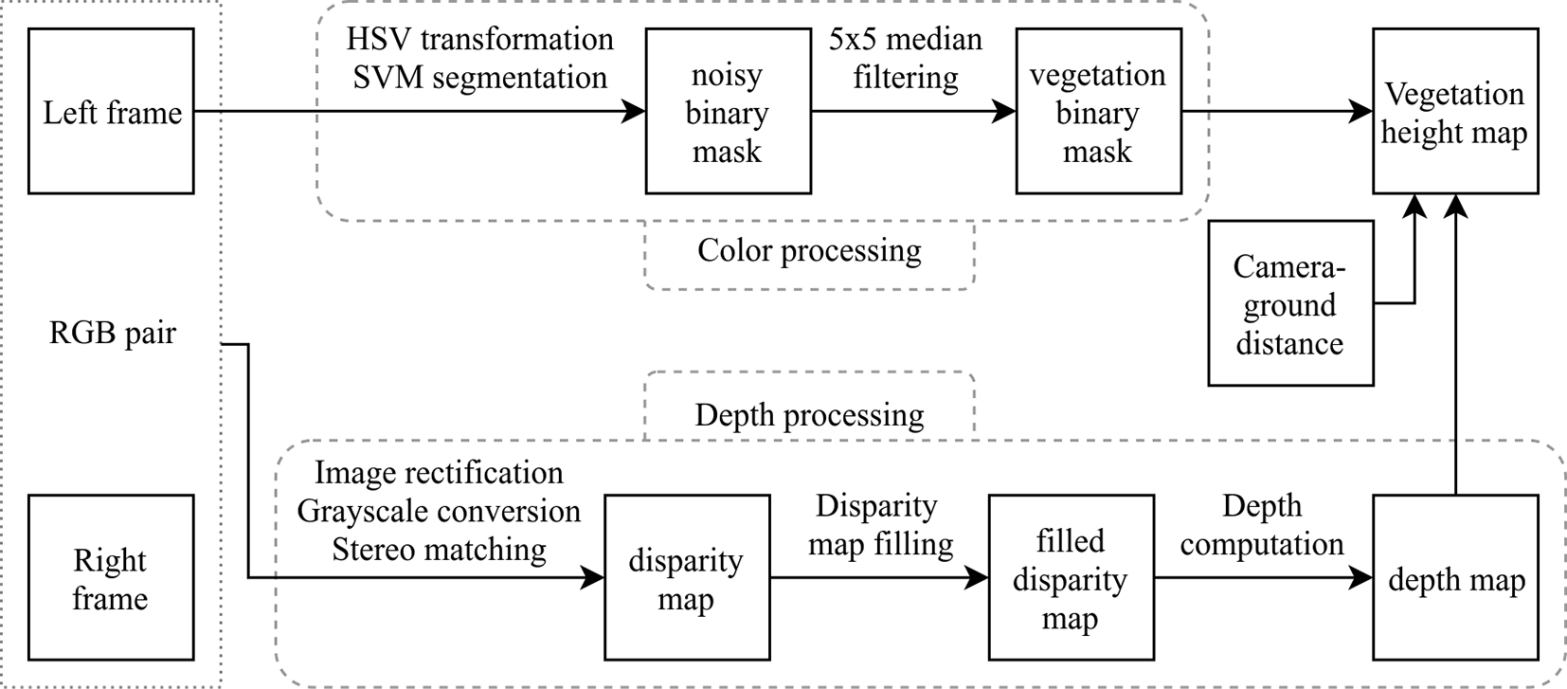
Processing pipeline.

**Figure 3:**
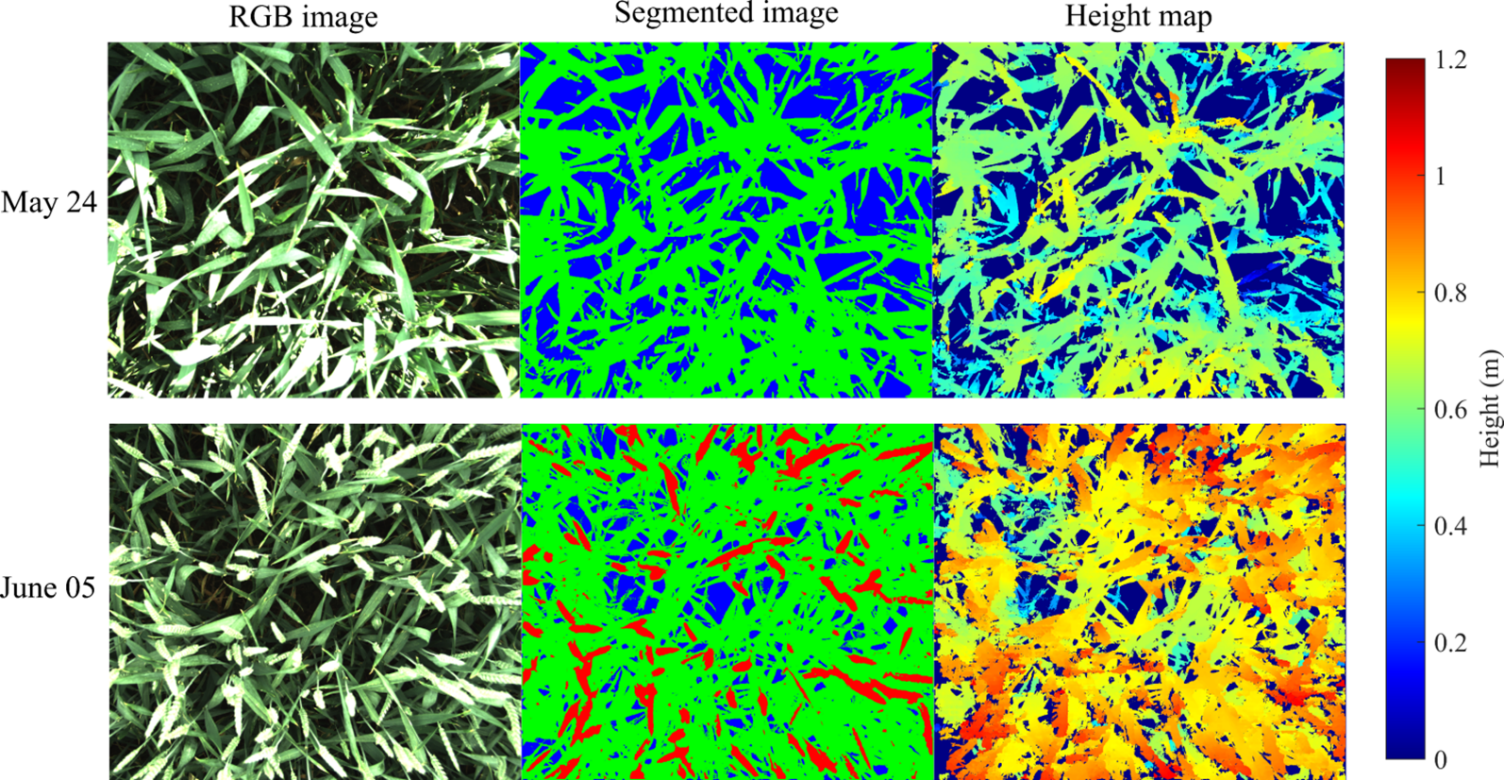
RGB image, segmented image (soil is blue, leaves are green and spikes are red), and height map for soil-leaves image (May 24) and soil-leaves-spikes image (June 05).

To extract only depth of plant objects, the segmentation mask was applied on the depth map. Ground-wheat distances (plant heights) were computed on the basis of this plant depth map. Plant heights are simply the difference between camera-wheat and camera-ground distances. The canopy height can be estimated by different descriptors such as the median, the 75th percentile, the 95th percentile, and the standard deviation of the ground-wheat distance. **Figures 2** and **3** show the image treatment pipeline from color images to height maps of plant elements.

### LAI and MTA Estimation by Global Delaunay Triangulation

The computation of LAI and MTA was based on geometric operations. Using the stereo calibration parameters and the height mapping, a 3D point cloud was generated for each image pair. The coordinate system of the point cloud was centered on the left camera and the z-axis was parallel to the optical axis of the system. The xy-plane of the system was theoretically parallel to the ground. The 3D point cloud was converted to a 3D mesh by means of a Delaunay triangulation. This process identified each point of the newly generated mesh as a vertex, and created associations between neighboring points in the form of edges. The resulting mesh was made of triangular faces formed by these vertices and edges. A size criterion was used to delete unnatural giant triangles, formed by neighbor points belonging to different leaves. Considering a triangular face with vertices ABC, the area was computed as half of the module of the cross product of two edges of the considered face, and reads

$$A_{triangle}=\frac{\left\|\vec{AB}\times \vec{AC}\right\|}{2}$$

The total plant area *A_plant_* was the sum of the areas of the individual triangles. The soil area below these plants was computed as follows

$$A_{soil}=\frac{{\bar{Z}}_{W}\cdot P_{S}\cdot A_{image}}{f_{m}}$$

where ${\bar{Z}}_{W}$ is the average camera-wheat distance (m), *P_S_* is the size of a pixel side on the sensor (5×10^-6^m), *f_m_* is the focal length (m) and *A_image_* is the area of the image (pixel). Finally, the LAI was the ratio between *A_plant_* and *A_soil_*. The tilt of each triangle was defined by

$$\theta_{triangle}=acos\frac{{\left(\vec{AB}\times \vec{AC}\right)}_{z}}{\left\|\vec{AB}\times \vec{AC}\right\|}$$

MTA was computed as the average tilt of the vegetative triangles over a stereo image without regard to the orientation of the triangles. The assumption was made that all triangle azimuths were equiprobable, which is verified for many crop canopies (Weiss et al., 2004).

### MTA and LAD Estimation by Local Fitting

**Figure 4:**
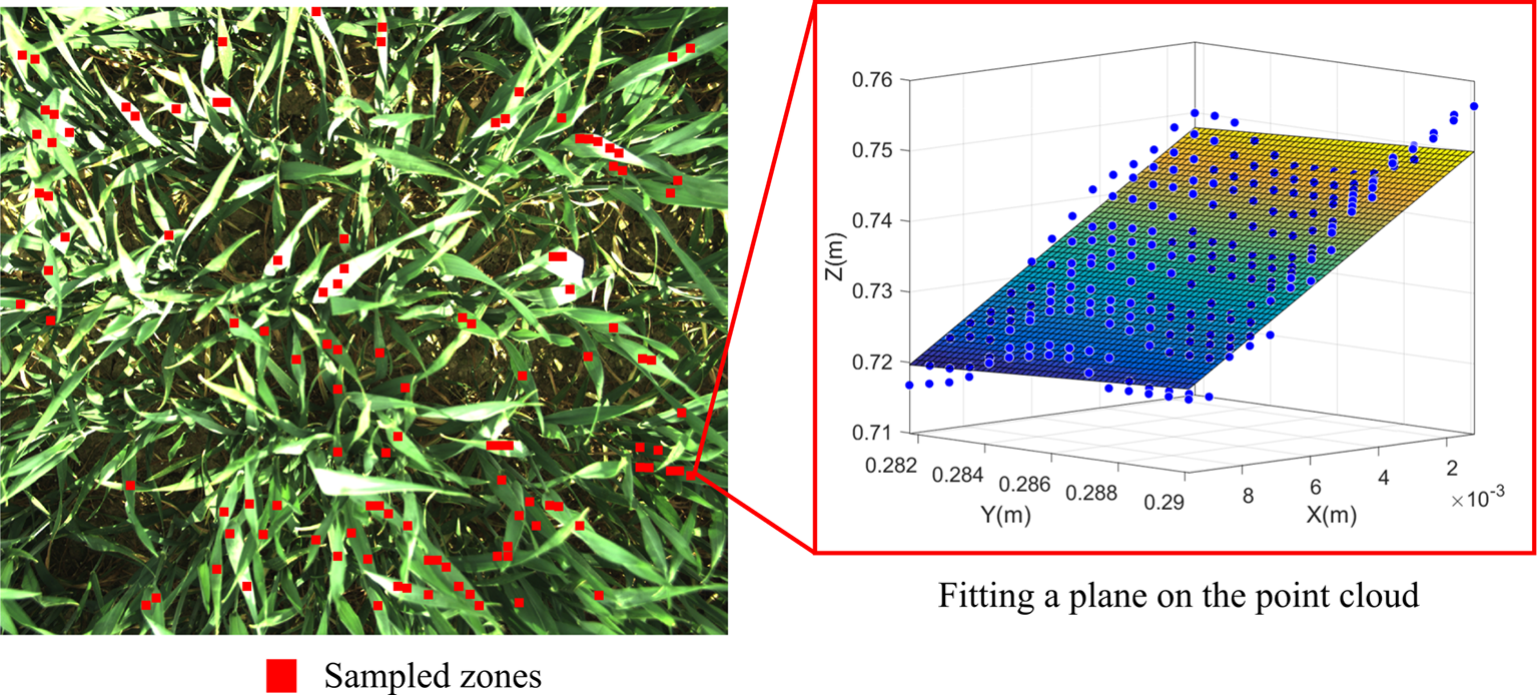
Local fitting of leaf surface: (left) sampled leaf zones, (right) fitted plane on the point cloud associated to a leaf zone.

Another method is proposed to provide an estimation of LAD in addition to MTA, one that would present greater robustness to noise and incompleteness in the 3D frames. An autonomous algorithm was developed to systematically select regions of interest (ROIs) in images, as opposed to other practices consisting of interactive selection of ROIs by human intervention (Biskup et al., 2007). Such manual operations are rendered virtually impossible in this study by the abundance of images in the dataset and the abundance of leaves in each image (**Figure 4**). The original approach is based on the sampling of non-overlapping leaf zones for which the height of each pixel can be computed. Leaves edges are identified by means of an edge detection Canny filter so as to avoid a zone covering different leaves. A first iteration is performed by selecting leaf zones of 30 × 30 pixels, corresponding to approximately 70 mm² for 2560 × 2048 pixel images. The algorithm searches for non-overlapping ROIs satisfying strict quality criteria: all pixels have to present a plausible height value and the zone cannot contain any detected edge. If the number of zones satisfying all these criteria is too small, the algorithm starts over the process by searching for smaller zones such as 20 × 20 pixels and finally 10 × 10 pixels. For each ROI, a plane is adjusted on the associated point cloud and the tilt angle of this local leaf face is deduced from the normal to the plane. MTA is computed as the average of the tilt angles of the sampled ROI over a stereo 3D frame. LAD is obtained by recording the frequencies of those tilt angles for 5° classes.

## Results and Discussion

### The Stereo Matching Algorithm

Stereo matching is a challenging task. The projections of an object on two different optical planes are not necessarily represented by the same number of pixels. This results in incomplete pixel-to-pixel matching for depth computation. Visual occlusions can also prevent full depth mapping of stereo-images.

The SGBM algorithm was firstly assessed on the Middlebury dataset, which contains reference images provided with dense disparity maps (Scharstein and Szeliski, 2002; Scharstein and Szeliski, 2003; Scharstein and Pal, 2007). The *cones* and *teddy* reference images were firstly considered due to their complex scenes with contrasted objects. The stereo matching was performed with errors of 7,4 % for *cones* image and 9,5 % for *teddy* image. For less complex reference images, the error significantly decreased to 2 %. Finally, the algorithm was tested on *Aloe* reference image that is the most representative image of vegetation and led to an error of 8,4 %. It is noticed that this error represents the number of pixels for which disparities differ from at least one pixel. It means that disparities differing from one pixel contributed to the error, with the same weight as a more important error. In comparison with the literature (Scharstein and Szeliski, 2002) and more particularly with an optimized stereo matching algorithm leading to errors of 2.9 % and 7 % for *cones* and *teddy* images respectively (Li et al., 2017), the performances of the SGBM algorithm on the Middlebury dataset were considered as sufficient.

**Figure 5:**
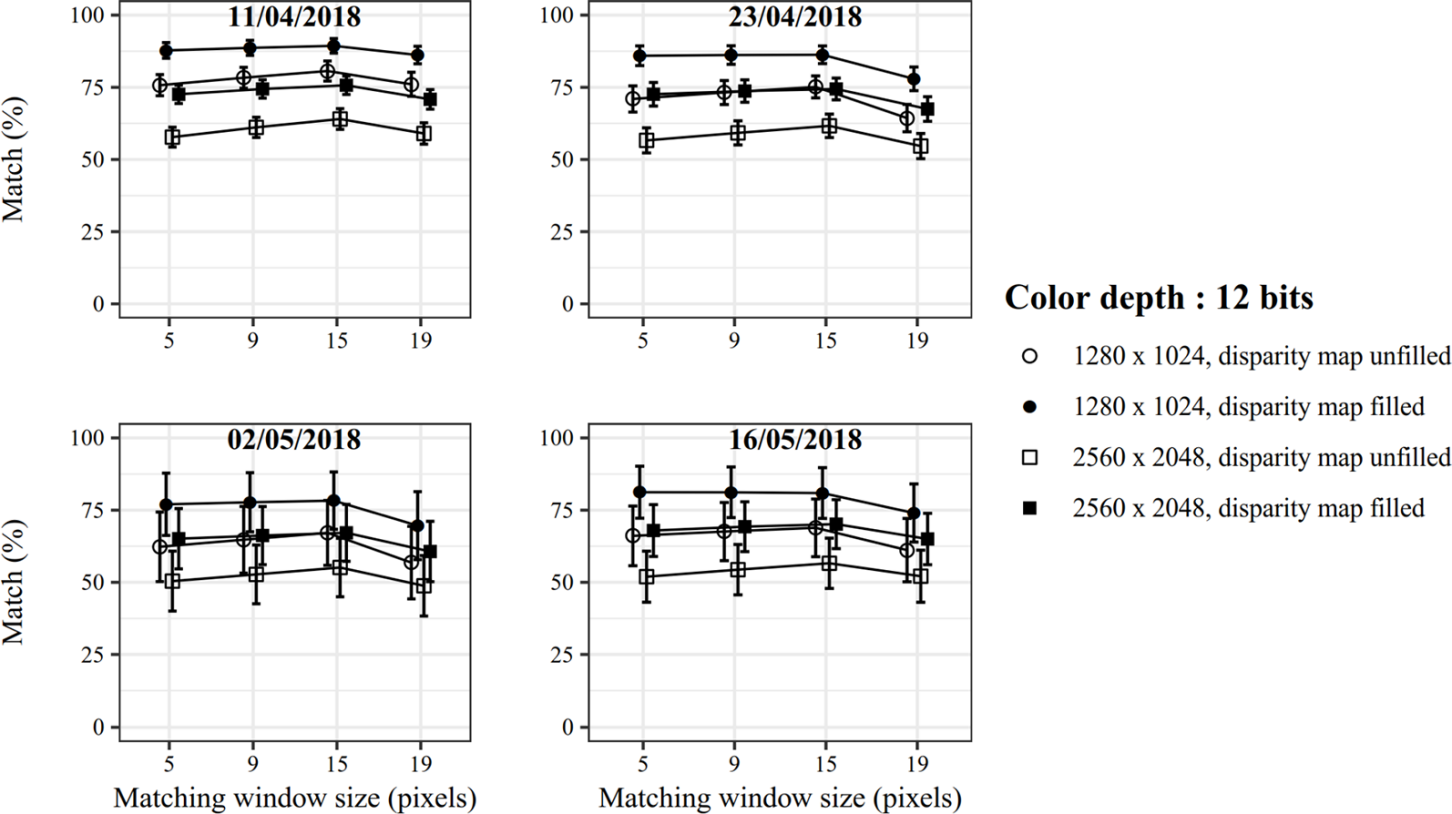
Stereo matching performance for 12-bit images.

**Figure 6:**
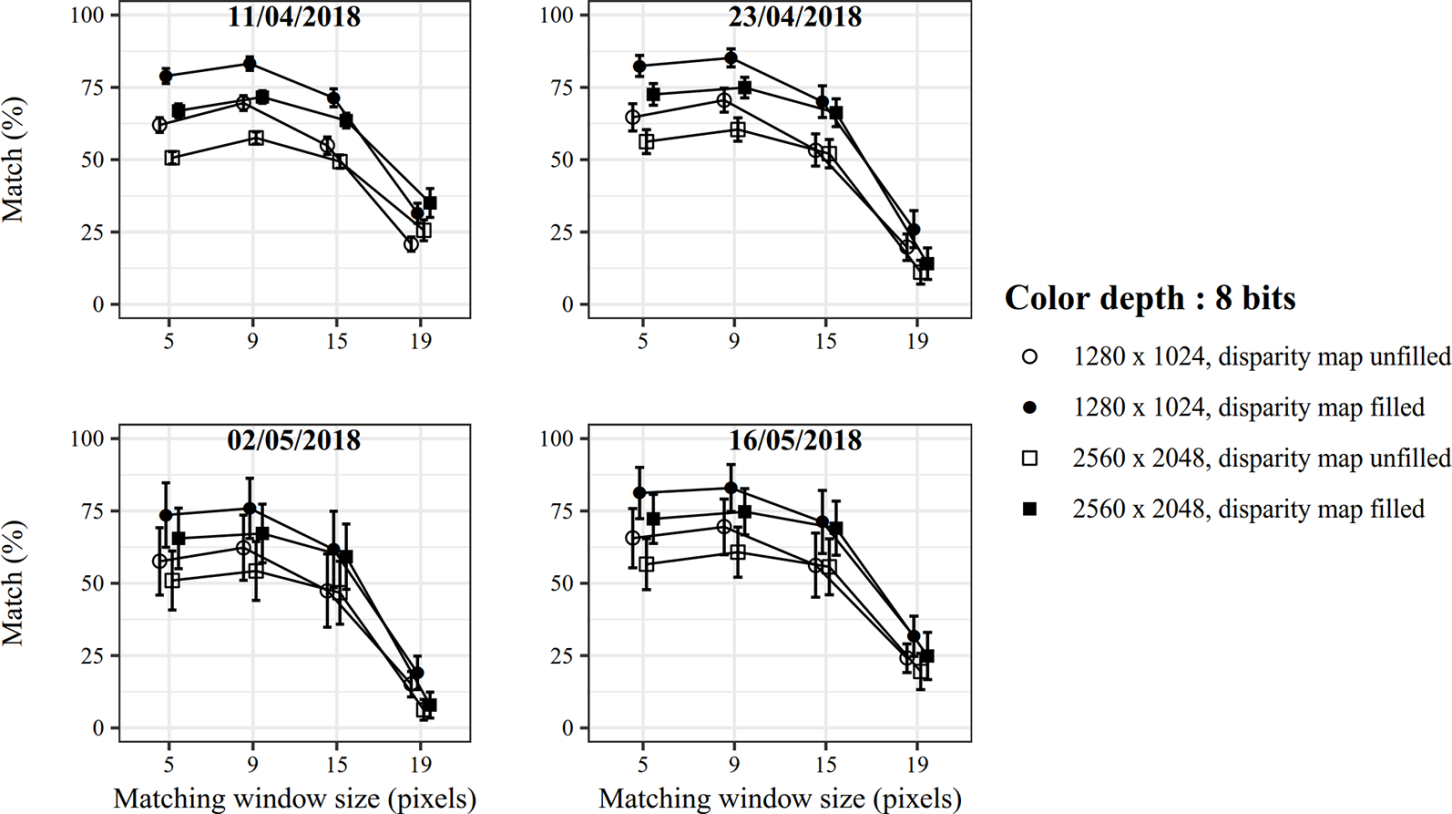
Stereo matching performance for 8-bit images.

Secondly, stereo matching performances were evaluated for the specific case of winter wheat canopy by studying the effects of image size, pixel color resolution, disparity map filling, and matching window size on images acquired at four dates (**Figures 5** and **6**). Since no reference maps were available for the canopy images, an indicator based on the plausible height percentage was introduced to assess the matching quality. This indicator expresses the proportion of plant pixels for which the computed height value ranges between the ground and 0.6 m below the stereoscopic device, even though this height value may be inaccurate. This choice is based on the hypothesis that the highest plants may have been found 0.6 m below the cameras, considering that the average camera-wheat distance was approximately one meter but that some plants were taller than the average canopy level. As shown in **Figures 5** and **6**, this sensitivity analysis revealed that the best stereo matching performances are obtained for an image size of 1280 × 1024 pixels with a color resolution of 12 bits. Moreover, the computation time to extract the disparity map was roughly ten times higher for 2560 × 2048 pixels images than for 1280 × 1024 pixel images. The absolute value of the computation time depends on the hardware. As an order of magnitude, the average time to compute a disparity map for 1280 × 1024 pixel images was around 0.8 seconds on a Windows computer with a 2.8 GHz Intel Core I5-4200H processor. This computation time was not significantly influenced by color resolution. Applying an interpolation-based filling algorithm helped to complete the disparity map. This step does not necessarily bring reliability to the results but is required to compute traits such as leaf surface where a dense 3D point cloud is crucial to properly adjust a mesh. On the contrary, a dense 3D point cloud is not compulsory to extract canopy height and map filling becomes accessory. Regarding the matching window size, little effect was observed for 12-bit images. Overall, a window size of 15 pixels provided the best results regardless of the image size. For 8-bit images, the choice of a proper matching window size was more decisive. The optimum for wheat images was found for a size of 9 pixels. It is noted that the stereo matching variability increased at the last two dates. As more images were acquired at those dates to take into account the multiplication of fertilization practices, the acquisition was spanned over a longer period which could explain more variability in the matching due to varying illumination conditions.

### Effect of Direct Sunlight on Matching

**Figure 7:**
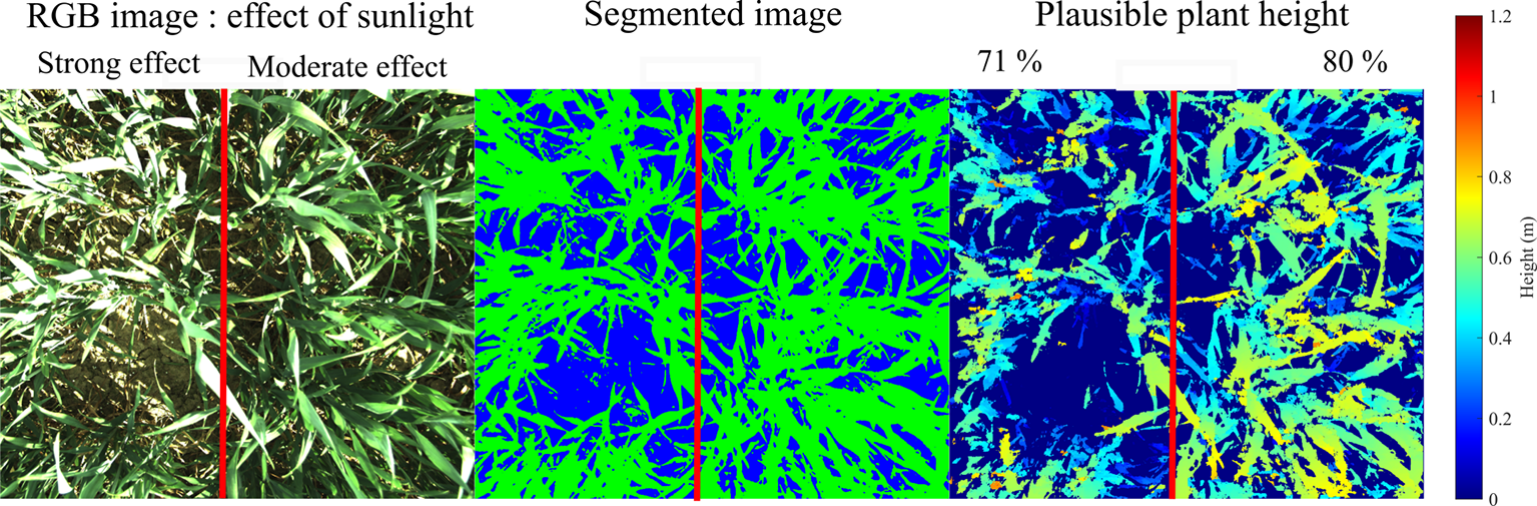
Effect of direct sunlight on segmentation and stereo matching for two zones of a same image (May 16). The left part of the image present 9.1 % of saturated gray-level pixels while the right part only contains 2.2 %.

Commonly used stereo matching algorithms present a Lambertian constraint which specifies that the intensity of the projection of each point in an image must be independent of the angle with which the camera observes this point (Devernay, 1997). In the field, direct sunlight combined with reflection properties of leaves cause the non-respect of this constraint. As a result, pixel intensities of same points of the scene can be different in the two images of the stereoscopic device. This causes trouble for stereo matching in some zones exposed to direct sunlight, as illustrated in **Figure 7**. Moreover, such a light can reduce visible leaf texture (Müller-Linow et al., 2015). For these reasons, cloudy conditions better suit to image acquisition. A solution to increase robustness to sunlight would consist in improving the stereo matching algorithm by transforming the intensities of pixel neighborhoods in the matching process by means of the census transform (Zabih and Woodfill, 1994). Another possibility would be to avoid direct sunlight by using a shadowing device. This latter option has not been implemented for this study because the goal was to test an acquisition device as compact and polyvalent as possible.

The effect of sunlight must be taken into account for the design of the stereoscopic acquisition system, especially for the baseline and camera height sizing. For this study, a baseline of 50 mm was used. For the same distance between the cameras and the plants (1 m), Li et al. (2017) have shown that a baseline of 80 mm provides the best compromise between depth accuracy and mismatch rate. However, their test took place in indoor conditions. Increasing the baseline could increase the damaging effect of sunlight in the field since the cameras would observe a same point with a more important angle difference. Moreover, a baseline increase induces a reduction of the overlap between the left and right images. This could be an inconvenient to perceive the variability among plants in the field. For some applications, a larger distance could be chosen. This could reduce the effects of distortion and increase the number of plants captured at one shot. Nevertheless, the baseline should probably be adapted as its choice depends on the measurement distance.

### Segmentation Robustness to Light Conditions

In field conditions, the development of a segmentation method that is robust to environmental conditions (light, wet or dry soil, shadows, dead leaves on the ground) is a challenge. The proposed method, based on machine learning and transformation in HSV components, performed soil-leaves segmentation with an accuracy of 98.5 % for the validation dataset. By adding depth and texture information, the method separated soil, leaves and spikes with an exactitude of 99.8 % on the validation dataset. Such performances were however overestimated due to pixel saturation. Those pixels are either ground, leaves, or spikes but, as the intensity values peak, their classification is impossible without using depth information. For the sake of properly training the classifier, training zones in saturated areas have only been selected for the most commonly saturated class (leaves for the soil-leaves classification and spikes for the soil-leaves-spikes classification). As a result, badly classified pixels in saturated zones (*e.g.* saturated soil pixels classified as leaves pixels) could not be taken into account to compute the classification error, leading to an overestimated accuracy.

Based on this consideration, it is suggested to implement an auto-exposure acquisition algorithm to mitigate image saturation. Such algorithm would have to reduce the integration time if the image comprises more than a certain percentage of saturated pixels. This threshold has to be chosen carefully in order to keep benefit of the color resolution. Preliminary tests indicated that a saturation threshold of 3% remains acceptable for images containing spikes, which are most susceptible to cause saturation in images.

### LAI Measurement: Stair-Step Effect and Overlapping Leaves

**Table 1** shows the results of surface area measurement in laboratory on three non-curved leaves of known area arranged in 20 different positions. The random error is the average difference between the measurements and the mean of the measurements, expressing the precision. The systematic error, related to the accuracy, is the average difference between the measurements and the reference value. Close area values were found for the 20 positions but the area was systematically overestimated. This is caused by stair-step faces in the triangulation process in comparison with the real smooth surface. This stair-step effect is due (i) to random errors in depth measurement and (ii) to the resolution of depth measurement itself, which means that even without random errors the reconstructed surface would present a stair-step shape. Indeed, for most plant elements, minimal depth resolution is close to the distance represented by one pixel (near 0.5 mm for 1280 × 1024 images at 1 m distance between camera and observed objects). As a result, angles of triangles are either 0° or superior to 45°, which creates the stair-step effect on the reconstructed surface. This systematic bias leads to intrinsically overestimated area measurement. **Table 1** presents the effect of median filtering of depth maps on this phenomenon.

**Table 1:** Accuracy and precision of leaf area measurement determined in laboratory.

	2560 × 2048 pixels			1280 × 1024 pixels		
	No filter	Median 5 × 5	Median 10 × 10	No filter	Median 3 × 3	Median 5 × 5
**Area: systematic error**	82.27%	66.17%	48.70%	58.47%	55.88%	51.12%
**Area: random error**	7.40%	5.70%	4.40%	4.65%	4.40%	4.34%

The stair-step effect was more important for 2560 × 2048 than for 1280 × 1024 images as already emphasized by Leemans et al. (2013) with images of 1280 × 960 and 1024 × 768 pixels. A minimal image size is however necessary to distinguish plant details, take into account leaves curvature and thin leaf parts. The number of pixels to use depends on the distance between the canopy and the cameras and should be properly chosen.

**Figure 8:**
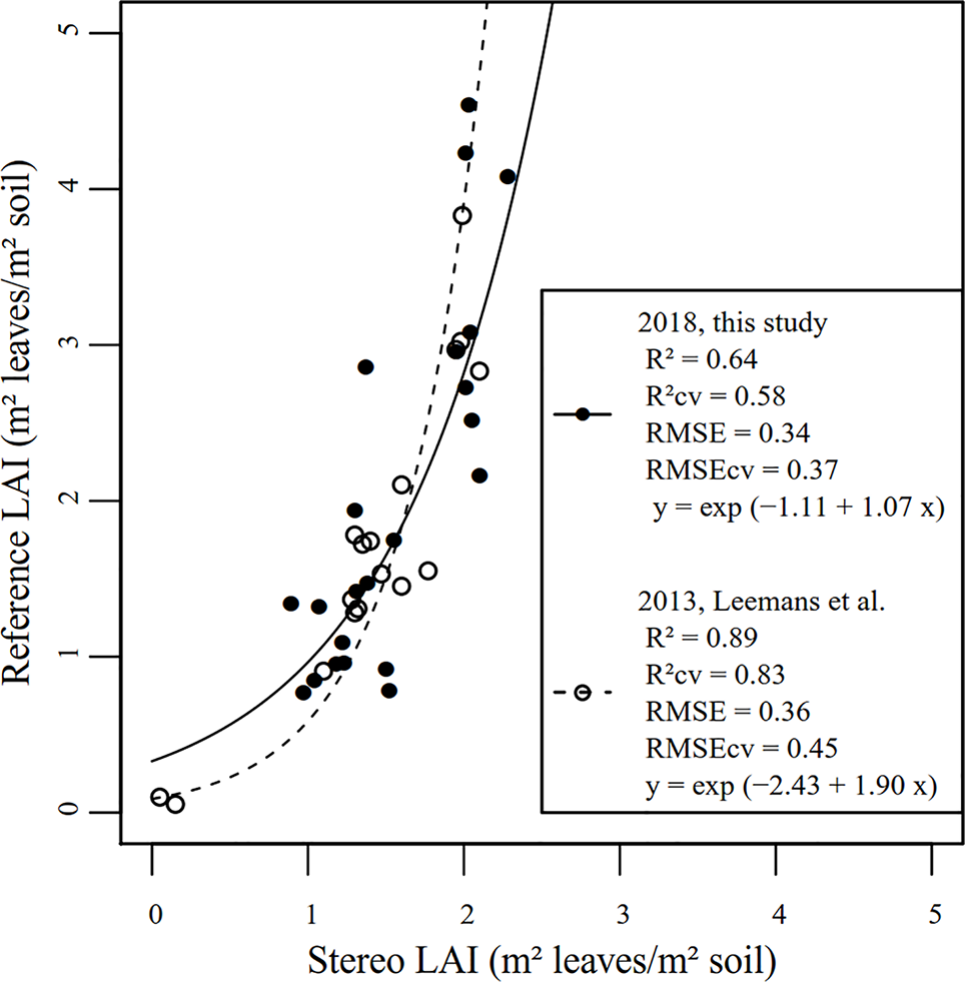
Relation between manual reference measurements and LAI measured by stereo vision for the 2018 data and for the data acquired by Leemans et al. (2013).

Stereo LAI measurements in the field were impacted by two phenomena: (i) the stair-step effect tending to overestimate the measured area and (ii) overlapping leaves tending to underestimate the measured area. As a result, absolute measurement was inaccurate and a calibration curve was necessary. The best model to directly fit stereo LAI with manual measurement was an exponential regression (**Figure 8**). Similar results were highlighted by Leemans et al. (2013). Such evolution can be explained by the canopy structure dynamics. For the first development stages (LAI < 2), a linear relation would have been more appropriate but as the crop grew (LAI > 2), the canopy became denser and many leaves overlapped. As a result of this complex canopy structure, it appears that the model may lack robustness since it fails to take into account leaf surfaces of the lower vegetation stratum. Indeed, stereo vision essentially records the leaf area of the upper foliage stratum. A suggestion for a further study implying stereo LAI measurement would be to follow the dynamics of the area of the upper stratum of a same crop zone at each stage and integrate it over the whole season. The model would take into account area of the upper stratum as well as area of lower leaves previously measured to extract LAI at each stage. The height information would be useful to distinguish foliage floors.

As reference LAI measurements are time-consuming, all data acquired in 2013 by Leemans et al. and all data acquired in 2018 were used to calibrate the models. A leave-one-out cross-validation method was applied to estimate the errors of both models. The drawback of cross-validation is that validation and calibration data might not be independent. However, the independent 2013 dataset cannot be used to externally validate the model developed in this study because the acquisition system set-up and the algorithms were improved in the meantime. Despite those differences, the two studies, performed on different plots, dates, and years provided pretty close results. The 2018 modifications actually helped to reduce the root mean square error (RMSE). The higher coefficient of determination (R²) in 2013 is explained by the measurement of two micro-plots at early growth stage leading to reference and stereo LAI close to zero.

Destructive manual LAI measurements were performed on 0.07 m² areas (0.5 m of one crop row, with 0.14 m spacing between rows) while images represented nearly 0.5 m² zones. As a result, stereo measurements were more suitable to take into account intra-plot variability. Manual reference measurements may have been realized in local spots non representative of the whole micro-plot. This aspect has to be considered when assessing the quality of the regression models. As a perspective for further research, a practical solution to increase the spatial extent of reference LAI measurements and to accelerate them would be to exploit the high correlation between leaf weight and leaf area, as performed by Roth et al. (2018). Finally, as highlighted by Baret et al. (2010), the definition of the measured variable is important. The imaging method recorded the area of all the green elements while only leaves were manually sampled. Nevertheless, due to the nadir position of the cameras, few stems were visible on the images and their contribution was considered negligible. Should the orientation of the acquisition device change, thus making more stems visible, the considered variable should rather be the green area index, which takes into account all the green elements and not only leaves.

### The Effect of Wind on LAI Measurement

In field conditions, wind is susceptible to disturb the measurement, especially by inducing blur in the stereo images and variability between acquisition sequences. In order to assess the measurement repeatability, five pairs of images of the same zones were acquired at 15 seconds of interval. The coefficients of variation (ratio between mean and standard deviation) for the LAI measurement are presented in **Table 2**. Overall, the wind-induced variability was rather small, except on April 23. The average wind speed measured for each date does not allow explaining this higher variability. Based on visual in-field observations, it is suggested that the variability depends on the development stage. On April 09 and 11, at tillering, plants were low and hardly impacted by wind. On April 23, at Zadoks stage 31 (Zadoks et al., 1974), the stems were erected but the canopy was not dense. Plants organs were moved by wind which caused variability in LAI measurement. However, from April 30, the canopy was denser so the impact of wind on LAI measurement was diminished. Indeed, canopy flow is influenced by vertical profile of plant density, canopy height, and element flexibility, which depend on the development stage (Cionco, 1972). The conclusion that wind has little effect on the imaging method is only valid if the two images are acquired simultaneously, as performed in this experiment. As highlighted by Kaczmarek (2017), if the acquisition of the two frames is not simultaneous, even minor wind will highly decrease stereo matching performances.

**Table 2:** Coefficients of variation (CV) of LAI measurements for a same zone captured at 15 second intervals.

Date	April 09	April 11	April 23	April 30	May 02	May 16
**CV (%)**	0.3	0.6	11.9	3.2	1.8	2.5
**Average wind speed (m/s)**	3.8	1.4	4.9	6.7	5.5	5.6

### MTA and LAD Measurement

**Figure 9:**
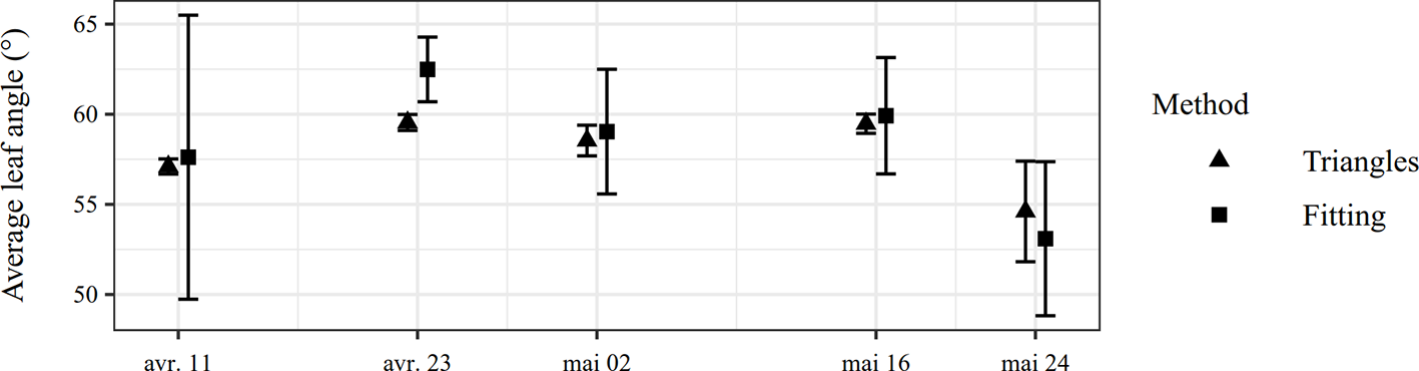
Comparison between Delaunay triangulation and local fitting methods to estimate MTA in field experiments. Vertical bars indicate the standard deviation of the MTA estimation for the different images acquired at a same date.

MTA measured in laboratory are reported in **Table 3**. For tilted surfaces in the images, the stair-step effect does not cause an important systematic error for the Delaunay triangulation method because angles of the triangles are favorably averaged, i.e. the error on all inclination angles gets canceled out. The same conclusion has been found by Leemans et al. (2013). However, for a flat horizontal surface (tilt angle = 0°), some triangular faces are tilted due to depth estimation errors but, as their inclination is not signed, they cannot compensate for each other to obtain on average a 0° angle. As a result, the average angle is necessarily overestimated. To confirm this hypothesis, the error was computed only for the reference leaves showing an angle superior to 15°. In this condition, the error significantly decreased, which indicated that nearly flat surfaces were sources of important errors. The use of a median filter on the depth map helped to reduce the stair-step effect. In all cases, the local fitting method gave better results than the Delaunay triangulation method. However, this conclusion is limited to the case of straight leaves without overlaps. For a real canopy, the Delaunay triangulation method might be more adapted to take into account leaf curvature and leaf overlaps. Moreover, the Delaunay triangulation method considers all the pixels while the local fitting approach only focuses on some zones. In the field, using 1280 × 1024 images, MTA ranging from 53° to 62° were recorded for the different acquisition dates and fertilization practices (**Figure 9**). The variability of MTA determined by the Delaunay triangulation method ranged from 0.74% to 1.45% for the different dates, which underpins that the method is precise. The variability was higher for the local fitting method (**Figure 9**).

**Table 3:** Average absolute errors on MTA measurement on reference leaves with 10 inclinations (ranging from 0 to 75° relative to a plan perpendicular to the optical axis of the cameras) computed in laboratory for different angle computation methods and image treatments (pixel binning and median filtering of the depth map).

	2560 × 2048 pixels			1280 × 1024 pixels		
	No filter	Median 5 ×5	Median 10 ×10	No filter	Median 3 ×3	Median 5 ×5
**Delaunay triangles**	11.65°	9.09°	6.37°	7.37°	7.12°	6.16°
**Local fitting**	4.00°	3.98°	3.81°	3.51°	3.46°	3.55°
**Delaunay triangles** **angles >15°**	7.30°	6.87°	6.31°	4.65°	4.76°	4.41°
**Local fitting** **angles >15°**	4.25°	4.27°	4.20°	3.36°	3.32°	3.42°

**Figure 10:**
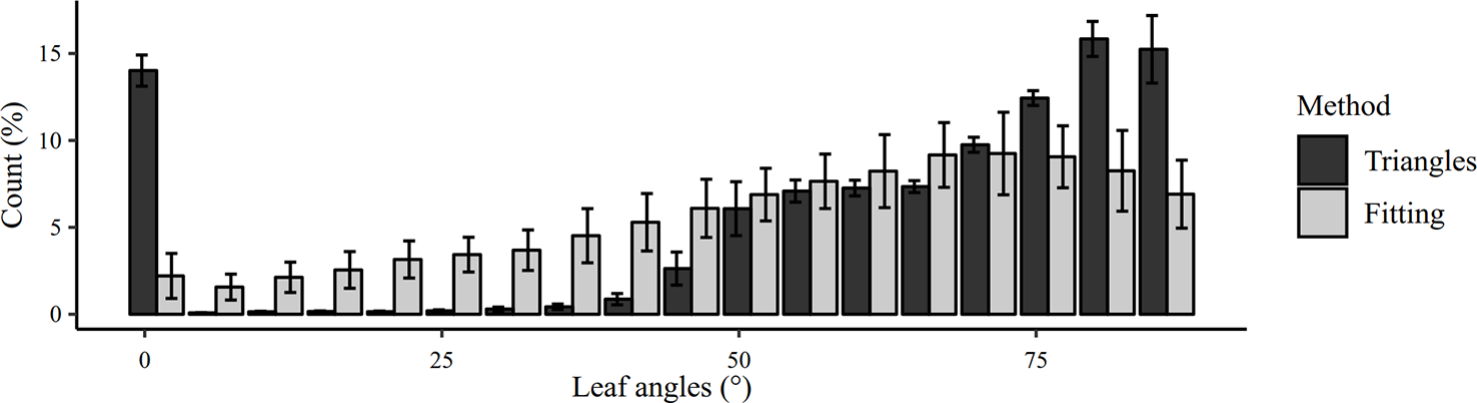
Comparison between Delaunay triangulation and local fitting methods to estimate LAD for images acquired on May 02. The graph shows the frequency and the standard deviation for each 5° class.

The Delaunay triangulation method seems the most precise to measure MTA. However, this method failed to provide LAD (**Figure 10**). Due to the stair-step effect, triangles were either horizontal or tilted with an angle superior to 45°. This was not a problem to measure an average angle but the inclinations of these triangles could not be used to study the angle distribution of larger leaf elements. This drawback is corrected by the local fitting approach. By considering the angles of the planes adjusted on some leaf faces, it was possible to get a good overview of the angle distribution of leaf faces. The obtained average LAD was very similar for the different dates. A potential improvement of angle measurement would be to find a criterion to record the azimuth angle of the leaf segments (triangles or fitted planes). It would allow to verify the assumption that all azimuth angles are equiprobable for the wheat plots considered. Indeed, for a sugar beet crop, Müller-Linow et al. (2015) have found that the azimuth angle distribution of leaves was not uniform. They also noticed that the preferential orientation of leaves changed over the season.

MTA and LAD were not manually measured in the field. Due to wind, canopy structure and leaf curvature, a direct manual measurement is very difficult, time-consuming and unreliable, hence the interest of automated measurement that relies on small leaf segment to take into account leaf curvature. As a result, no reference values were available but MTA values computed by two different and independent methods were close, suggesting that both measurements could be relevant. In comparison, Shibayama and Watanabe (2007) measured MTA between 56° and 65° for two different wheat varieties using polarized light and LAI-2000 sensors. Hosoi et al. (2009) measured MTA between 44° and 56° for different dates by using a LiDAR. However, their leaf segment selection method was not automatic and the variability of MTA measurement was around 40 %, against 1 % for the proposed method. These comparisons must be put in perspective. According to Yanli et al. (2007), the angle distribution widely depends on the wheat variety. Huang et al. (2006) found similar LAD by using canopy reflectance and characterized that kind of wheat variety as “erectophile”. According to de Wit (1965), the distribution presented in **Figure 10** may rather be classified as “plagiophile” because oblique leaves are more frequent than vertical leaves.

### Comparison of Stereo-Based and Manual Height Measurements Before and After Spike Emergence

**Figure 11:**
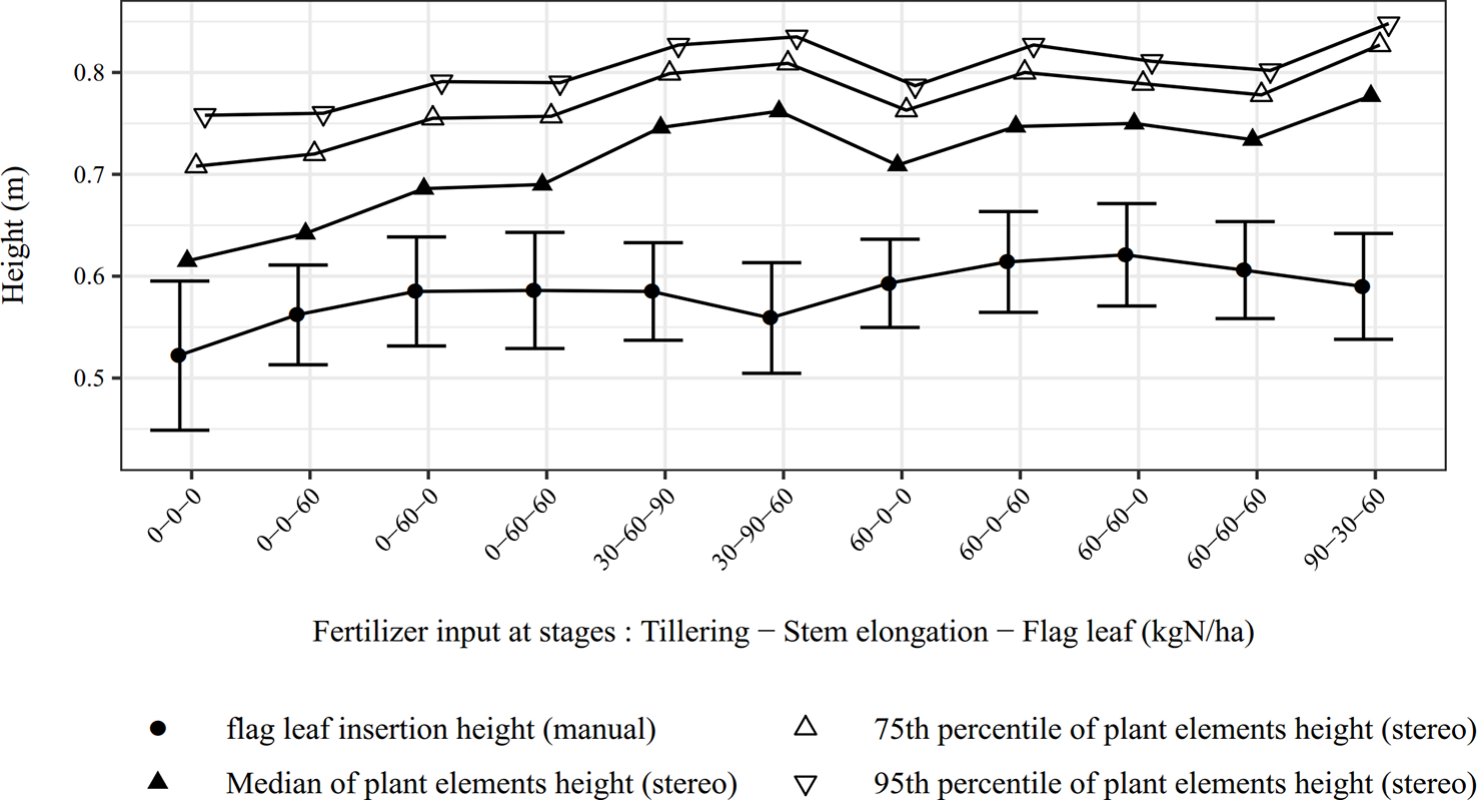
Comparison of manual and automatic measurements of canopy height for 11 micro-plots (May 24).

For data recorded before spike emergence, stereo-based and manual measurements with a meter stick provided non-equivalent indicators to describe canopy height. Both present advantages and inconveniences and should be used for different purposes. Manual measurements have the advantage that the operator directly chooses the point of interest (flag leaf tip, spike tip, last node) which is convenient to study specific vegetative organs. On the contrary, the image-based height measurement of specific points is a complex, and sometimes impossible, task due to the difficulties encountered to automatically detect such points (*e.g*: overlapping leaves prevent detection of nodes). For manual height measurement, numerous repetitions were necessary to obtain a robust estimation, which can be seen as the main drawback of this method. On the contrary, the stereo-based method allows acquiring height descriptors of a zone of several plants in a simultaneous way. As demonstrated by Cai et al. (2018), the height map yields complete height distribution which provides far more information on canopy development than a manual height measurement. Several statistical descriptors of the height deduced from the 3D point cloud are proposed in this study. The suggested descriptors are the median, the 75th percentile and the 95th percentile of the point heights. **Figure 11** shows the different height descriptors for both types of measurement. The comparison between the different fertilization practices revealed that global canopy height described by stereo vision seems a better indicator of cover development than the manually determined height of flag leaf insertion. A final note on height measurement at the vegetative stage concerns the manual reference method. A meter stick, as used for this study, is not the only possibility to record crop height. Using an herbometer (a plate of known weight attached to a rule), as described by Barmeier et al. (2016), would provide a weighted plant height. This measurement is considered to be more representative and objective that a measurement at a specific point. Moreover, as the herbometer measure a weighted height on a zone and not height at a point, it could be better suited to provide a reference for stereo vision. It would even be possible to design the herbometer with a size similar to that of the captured zone. A herbometer must however be adjusted to account for various degrees of stem stiffness, depending on growth stage or cultivar.

**Figure 12:**
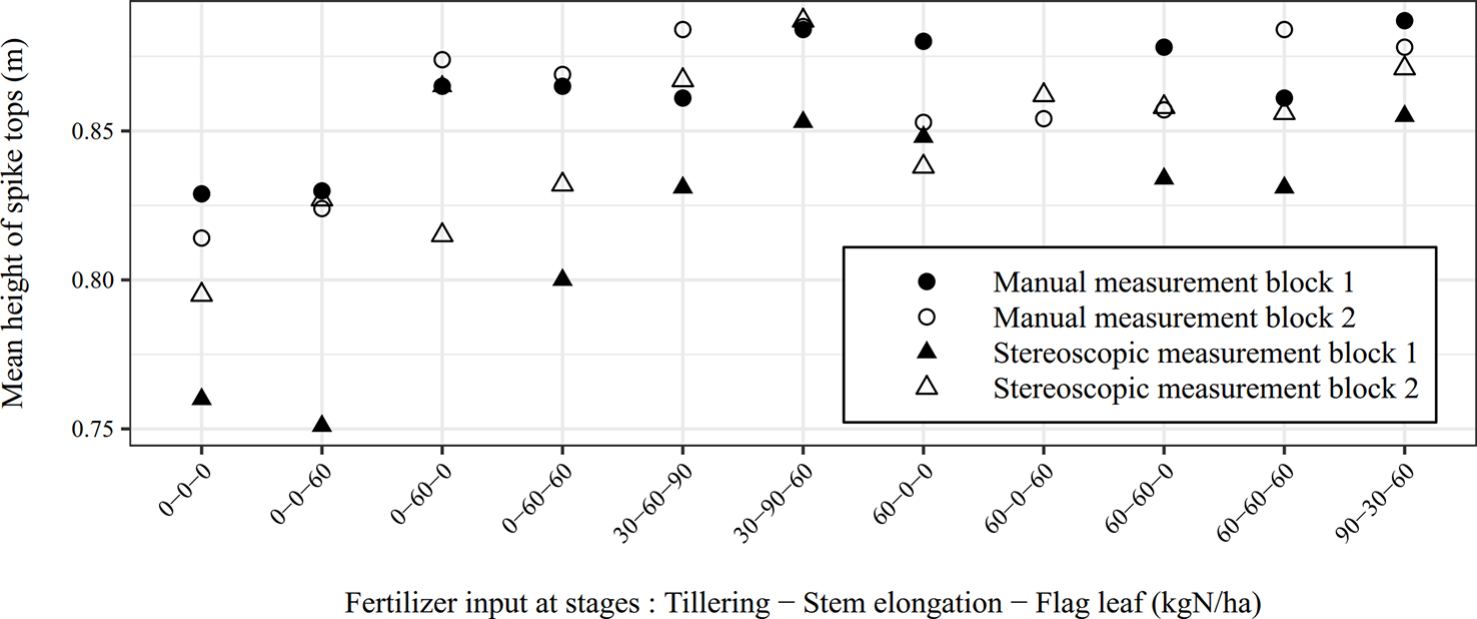
Comparison between the manual and the automatic measurements of the mean height of spike tops for two blocks of micro-plots (June 05).

For data recorded after spike emergence, the relevant height descriptor is the mean height of spike tips both for manual and imaging methods. For the automatic measurement, the height of each spike object was the 95th percentile of heights, so that the mean height of spike tips for one image was the mean of those 95th percentiles. This trait was measured for two blocks of micro-plots both manually and by stereo vision on June 05 (**Figure 12**). By considering the manual measurements as a reference, mean spike top heights were measured by stereo vision with an accuracy of 97.1% (RMSE of 0.016 m). For micro-plots of block 2, manually and automatically measured mean height of spike tops were close. For block 1, the automatic measurement systematically underestimated the mean height but the evolution of height according to the fertilization practices followed the same trend as for manual determination. The systematic error was not due to the accuracy of camera-spike distance measurements but may be due to some other issues such as saturated leaves badly classified as spikes and the determination of camera-ground distance. Those issues represent challenges inherent to field acquisition. As suggested above, a custom auto-exposure algorithm should help to deal with important image saturation. The second issue is more challenging. The camera-ground distance is not constant due to soil surface irregularities induced for instance by tractor passage. This problem could be obviously overcome by cumbersome manual measurements slowing down the image acquisition process. To avoid that, an estimation of the camera-ground distance can be deduced from the soil pixels depth. However, for dense and high canopies, the estimation of ground depth was not reliable due to the lack of visible soil spots rendering stereo matching troubles. Finally, because of an imperfect positioning of the acquisition device in the field, the cameras were not exactly perpendicular to the ground resulting in non-constant real camera-ground distance on the stereo image.

To conclude, stereo-based height measurement in a complex canopy offered an easy way to compare global canopy height and average spike top height of different micro-plots. However, the absolute height of micro-plots remained uncertain because of difficulties to automatically get camera-ground distance at each point of the area of interest. Manual measurements are useful to measure the height of specific plant elements that would be difficult to spot on images.

### Comparison With Other Proximal 3D Sensors

This section aims at answering the question: which 3D sensor to choose in order to measure wheat morphological plant traits such as LAI, foliar angle and plant height, by considering the literature updated with the results of this study? It focuses on the sensors that directly measure morphological features, and not on the sensors that rely on a relation between the architectural traits and reflectance. Several recent papers already compare the performances of the most common 3D sensors for high throughput plant phenotyping (Li et al., 2014; Vázquez-arellano et al., 2016; Perez-Sanz et al., 2017; Qiu et al., 2018; Wang et al., 2018). Based on these reviews, stereo vision is perceived as sensitive to sunlight and poorly adapted for outdoor imaging. However, this study demonstrates that stereo vision can be used for acquisition under natural conditions without any shadowing device and still provide dense depth information. The same conclusion cannot be drawn for methods that need to illuminate the scene such as time of flight cameras. To the knowledge of the authors of this paper, no recent study supports that a new generation of time of flight cameras would correct the issue of sensitivity to sunlight. Among the others methods, multi-view stereo and structure from motion can be considered as variants of a classic binocular stereo system. They have the potential to provide better results but necessitate to add cameras or to increase the number of shots of a same scene. Those methods should be envisioned instead of binocular stereo if the configuration of the acquisition platform allows it and if the amount of data to manage is not an issue.

Ultrasonic sensors are mentioned as a cheap solution to measure plant height. However, the wheat plants may not have sufficient density to reflect the echoes (Yuan et al., 2018). As a result, ultrasonic sensors sometimes do not really directly measure height at a plant surface. Moreover, they do not suit to reconstruct point cloud and fail to directly provide leaf inclination or area. To conclude, they should be chosen when the goal is to record canopy height but not for mapping the height of different organs or to construct a 3D point cloud.

At the moment, the real competitor of stereo vision for polyvalent 3D measurements in natural conditions is LiDAR. An important difference between stereo vision and LiDAR is that the latter directly provides 3D point clouds while stereo vision provides 2D height maps that can be converted into point clouds. Recent studies demonstrate that LiDAR can measure the morphological traits with performances equivalent or superior to those presented in this study for stereo vision. Jimenez-berni et al. (2018) measure height with a RMSE of 0.017 m. Leaf angle measurements could be obtained from LiDAR point clouds coupled with RGB image just as for stereo vision point clouds. Li et al. (2017) measure green area index with a RMSE of 0.22. However, even if LiDAR is more and more affordable, it remains costly and has to be coupled with a RGB camera to provide both morphological and color information. In addition, its use in outdoor conditions necessitates to deal with wind. To sum up, stereo vision may be preferred over LiDAR for the applications that need an inexpensive (around 2,000 euro for two cameras and objectives), compact (the two spaced cameras and their objectives form a device of around 0.1 **×** 0.1 **×** 0.04 m^3^) and polyvalent device that provides both color and morphological information without the necessity to fuse two sensors of different nature. Finally, stereo maps offer high spatial resolution while the spatial resolution of LiDAR measurements is conditioned by the scanning time and by its footprint.

## Conclusion

Stereo vision is a cheap, compact, and flexible way to study wheat canopy architecture in natural conditions. This is a polyvalent method allowing measurement of morphological traits such as LAI, MTA, LAD, and canopy or spike height. A stereoscopic vision system was set up to capture depth and color images of crop canopy. The acquisition system was calibrated and validated on winter wheat in an in-field nitrogen fertilization trial offering contrasting canopy architectures. LAI and MTA were computed based on a global Delaunay triangulation. It was shown that the image size might greatly affect the error. Median filtering of depth map helped to reduce the stair-step effect due to random errors in depth measurement and limited depth resolution. LAI was estimated with a cross-validation RMSE of 0.37 based on manual reference measurements. MTA was accurately estimated by the triangulation process. An original method based on a local surface fitting, was developed to properly extract LAD. Regarding the height measurement, the optical challenges faced to automatically measure camera-ground distance in dense canopies have been discussed and spike top height was measured with an accuracy of 97.1%. Overall, stereo vision provides 3D point clouds that allow precise comparisons of plots although the determination of absolute values of agronomic parameters such as LAI or canopy height might suffer from systematic errors.

Several solutions have been proposed to ease the development of the method in field conditions. Firstly, a robust segmentation method based on machine learning using HSV components helps to manage variable light conditions. Secondly, it has been shown that image size and color resolution can influence the stereo matching. A finer 12-bit color resolution was preferred to an 8-bit acquisition. For a camera-canopy distance of 1 m, 1280 × 1024 images presented better performances for stereo matching and LAI computation than 2560 × 2048 images. Thirdly, the wind had little effect on LAI measurement variability, except at stem elongation. It highlights that the development stage could be more important that the wind speed itself in terms of wind effect on the canopy. This observation should however be supported by testing multiple wind conditions at each stage before drawing a conclusion.

Perspectives are divided into two categories: (i) improving the stereo vision system and (ii) extracting supplementary traits from images. To improve the acquisition, a possibility would be to combine more than two cameras to build a multi-ocular system as proposed by Kaczmarek (2017) on small trees. This would yield more accurate and dense depth maps and help taking into account overlapping leaves. Another possibility would be to work with several pairs of cameras, observing the canopy with contrasting view angles. Concerning the other perspectives of trait extraction, the combination of depth, color, and texture information offers the potential to measure additional plant traits, especially at a smaller scale. The method could extract the morphology of yield-related organs such as flag leaves or spikes. It could also provide spike and seedling densities as well as proxies of tiller number.

## Data Availability Statement

The raw data supporting the conclusions of this article will be made available by the authors without undue reservation to any qualified researcher.

## Author Contributions

SD and BD contributed to establish the design of the experiment. AB, SD and VL contributed to the set-up of the stereo vision. SD acquired the data. SD and AB performed data analysis under the guidance of VL. The redaction of the paper was performed by SD, AB and BM. The study was motivated by BD and BM.

## Funding

The authors gratefully acknowledge the financial support provided by the Agriculture, Natural Resources and Environment Research Direction of the Public Service of Wallonia, Belgium (Project PHENWHEAT D31-1385). This publication also benefits from the support of the National Fund of Belgium FNRS-F.R.S. through a FRIA grant.

## Conflict of Interest

The authors declare that the research was conducted in the absence of any commercial or financial relationships that could be construed as a potential conflict of interest.

## Abbreviations

CMOS, Complementary Metal Oxide Semiconductor - *Camera sensor;* HSV, Hue Saturation Value - *Color space;* LAI, Leaf Area Index - *Plant trait;* LiDAR, Light Detection And Ranging - *Active 3D sensor;* MTA, Mean Tilt Angle - *Plant trait;* RGB, Red Green Blue - *Color space;* RMSE, Root Mean Square Error - *Indicator of regression quality;* ROI, Region Of Interest - *Area of interest on an image;* SGBM, Semi-Global Block Matching - *Stereo matching algorithm;* SVM, Support Vector Machine – *Classifier*.

## References

[B1] Andersen H. J. Reng L. Kirk K. (2005). Geometric plant properties by relaxed stereo vision using simulated annealing. Comput. Electron. Agr. 49, 219–232. 10.1016/j.compag.2005.02.015

[B2] Baret F. de Solan B. Lopez-Lozano R. Ma K. Weiss M. (2010). GAI estimates of row crops from downward looking digital photos taken perpendicular to rows at 57.5 zenith angle: theoretical considerations based on 3d architecture models and application to wheat crops. Agric. For. Meteorol. 150 (11), 1393–1401. 10.1016/j.agrformet.2010.04.011

[B3] Barmeier G. Mistele B. Schmidhalter U. (2016). referencing laser and ultrasonic height measurements of barley cultivars by using a herbometre as standard. Crop Pasture Sci. 67 (12), 1215–1222. 10.1071/CP16238

[B4] Biskup B. Scharr H. Schurr U. Rascher U. (2007). A stereo imaging system for measuring structural parameters of plant canopies. Plant Cell Environ. 30 (10), 1299–1308. 10.1111/j.1365-3040.2007.01702.x 17727419

[B5] Bréda N. J. J. (2003). Ground-based measurements of leaf area index: a review of methods, instruments and current controversies. J. Exp. Bot. 54 (392), 2403–2417. 10.1093/jxb/erg263 14565947

[B6] Bradski G. Kaehler A. (2008). Learning OpenCV (Sebastopol: O'Reilly Media, Inc.). 10.1109/MRA.2009.933612

[B7] Cai J. Kumar P. Chopin J. Miklavcic S. J. (2018). Land-based crop phenotyping by image analysis: accurate estimation of canopy height distributions using stereo images. PloS One 13 (5), 1–21. 10.1371/journal.pone.0196671 PMC5967702 29795568

[B8] Cionco R. M. (1972). A wind-profile index for canopy flow. Boundary-Layer Meteorol. 3 (2), 255–263. 10.1007/BF02033923

[B9] Constantino K. P. Gonzales E. J. Lazaro L. M. Serrano E. C. Samson B. P. (2015). Plant height measurement and tiller segmentation of rice crops using image processing. Proc. DLSU Res. Congress 3, 1–6. http://www.dlsu.edu.ph/conferences/dlsu_research_congress/2015/proceedings/FNH/021FNH_Samson_BPV.pdf.

[B10] de Wit C. T. (1965). Photosynthesis of leaf canopies. Agricultural Research Reports 663 (Wageningen: PUDOC).

[B11] Devernay F. (1997). Vision Stéréoscopique et Propriétés Différentielles Des Surfaces. [Thesis] (France: Institut National de Recherche en Informatique et en Automatique (INRIA)).

[B12] Dietterich T. G. Bakiri G. (1994). Solving multiclass learning problems via error-correcting output codes. J. Artif. Intell. Res. 2, 263–286. 10.1613/jair.10

[B13] Gibbs J. A. Pound M. French A. P. Wells D. M. Murchie E. Pridmore T. (2017). Approaches to three-dimensional reconstruction of plant shoot topology and geometry. Funct. Plant Biol. 44 (1), 62–75. 10.1071/FP16167 32480547

[B14] Hamuda E. Mc Ginley B. Glavin M. Jones E. (2017). Automatic crop detection under field conditions using the HSV colour space and morphological operations. Comput. Electron. Agr. 133, 97–107. 10.1016/j.compag.2016.11.021

[B15] He D. X. Matsuura Y. Kozai T. Ting K. C. (2003). A binocular stereovision system for transplant growth variables analysis. Appl. Eng. Agric. 19 (5), 611–617. 10.13031/2013.15308

[B16] Hirschm H. (2007). Stereo processing by semi-global matching and mutual information. IEEE Trans. Pattern Anal. Mach. Intell. 30 (2), 328–341. 10.1109/TPAMI.2007.1166 18084062

[B17] Hosoi F. Nakai Y. Omasa K. (2009). Estimating the leaf inclination angle distribution of the wheat canopy using a portable scanning LIDAR. J. Agric. Meteorol. 65 (3), 297–302. 10.2480/agrmet.65.3.6

[B18] Huang W. Niu Z. Wang J. Liu L. Zhao C. Liu Q. (2006). Identifying crop leaf angle distribution based on two-temporal and bidirectional canopy reflectance. IEEE Trans. Geosci. Remote Sens. 44 (12), 3601–3608. 10.1109/TGRS.2006.881755

[B19] Hui F. Zhu J. Hu P. Meng L. Zhu B. Guo Y. (2018). Image-based dynamic quantification and high-accuracy 3d evaluation of canopy structure of plant populations. Ann. Bot. 121 (5), 1079–1088. 10.1093/aob/mcy016 29509841 PMC5906925

[B20] Ivanov N. Boissard P. Chapron M. Valery P. (1994). Estimation of the height and angles of orientation of the upper leaves in the maize canopy using stereovision. Agronomie 2, 183–194. 10.1051/agro:19940305

[B21] Jay S. Rabatel G. Gorretta N. (2014).In-field crop row stereo-reconstruction for plant phenotyping, in: Second International Conference on Robotics and Associated High-Technologies and Equipment for Agriculture and Forestry (RHEA-2014), Vol. 10.

[B22] Jimenez-berni J. A. Deery D. M. Rozas-Larraondo P. Condon A. G. Rebetzke G. J. James R. A. (2018). High throughput determination of plant height , ground cover , and above-ground biomass in wheat with LiDAR. Front. Plant Sci. 9, 237. 10.3389/fpls.2018.00237 29535749 PMC5835033

[B23] Kaczmarek A. L. (2017). Stereo vision with equal baseline multiple camera set (EBMCS) for obtaining depth maps of plants. Comput. Electron. Agr. 135, 23–37. 10.1016/j.compag.2016.11.022

[B24] Kazmi W. Foix S. Alenya G. (2012).Plant leaf analalysis using time of flight camera under sun, shadow and room conditions, in: 2012 IEEE International Symposium on Robotic and Sensors Environments Proceedings.

[B25] Kise M. Zhang Q. (2008). Development of a stereovision sensing system for 3D crop row structure mapping and tractor guidance. Biosyst. Eng. 101 (2), 191–198. 10.1016/j.biosystemseng.2008.08.001

[B26] Leemans V. Dumont B. Destain M. F. (2013). Assessment of Plant Leaf Area Measurement by Using Stereo-Vision, in: 2013 International Conference on 3D Imaging (IC3D) pp. 1–5. 10.1109/IC3D.2013.6732085

[B27] Li L. Zhang Q. Huang D. (2014). A review of imaging techniques for plant phenotyping. Sensors (Basel) 14 (11), 20078–20111. 10.3390/s141120078 25347588 PMC4279472

[B28] Li D. Xu L. Tang X. Sun S. Cai X. Zhang P. (2017). 3D imaging of greenhouse plants with an inexpensive binocular stereo vision system. Remote Sens. 9 (12), 508. 10.3390/rs9050508

[B29] Lin T. T. Lai T. C. Liu C. C. Cheng Y. C. (2011). A three-dimensional imaging approach for plant feature measurement using stereo vision. Tarım Makinaları Bilimi Dergisi (J. Agric. Mach. Sci.) 7 (2), 153–158.

[B30] Müller-Linow M. Pinto-Espinosa F. Scharr H. Rascher U. (2015). The leaf angle distribution of natural plant populations: assessing the canopy with a novel software tool. Plant Methods 11 (1), 1–16. 10.1186/s13007-015-0052-z 25774205 PMC4359433

[B31] Pask A. Pietragalla J. Mullan D. Reynolds M. (2012). Physiological Breeding II A Field Guide to Wheat Phenotyping (Mexico : CIMMYT).

[B32] Perez-Sanz F. Navarro P. J. Egea-Cortines M. (2017). Plant phenomics: an overview of image acquisition technologies and image data analysis algorithms. GigaScience 6, 11. 10.1093/gigascience/gix092 PMC5737281 29048559

[B33] Piron A. Leemans V. Lebeau F. Destain M. F. (2009). Improving in-row weed detection in multispectral stereoscopic images. Comput. Electron. Agr. 69 (1), 73–79. 10.1016/j.compag.2009.07.001

[B34] Qiu R. Wei S. Zhang M. Li H. Sun H. Liu G. (2018). Sensors for measuring plant phenotyping: a review. Int. J. Agric. Biol. Eng. 11 (2), 1–17. 10.25165/j.ijabe.20181102.2696

[B35] Roth L. Aasen H. Walter A. Liebisch F. (2018). Extracting leaf area index using viewing geometry effects—a new perspective on high-resolution unmanned aerial system photography. ISPRS J. Photogramm. Remote Sens. 141, 161–175. 10.1016/j.isprsjprs.2018.04.012

[B36] Scharr H. Briese C. Embgenbroich P. Fischbach A. Fiorani F. Müller-Linow M. (2017). Fast high resolution volume carving for 3D plant shoot reconstruction. Front. Plant Sci. 8, 1680. 10.3389/fpls.2017.01680 29033961 PMC5625571

[B37] Scharstein D. Pal C. (2007). Learning conditional random fields for stereo. Proc. IEEE Comput. Soc. Conf. Comput. Vision Pattern Recogn. 1–8. 10.1109/CVPR.2007.383191

[B38] Scharstein D. Szeliski R. (2002). A taxonomy and evaluation of dense two-frame stereo correspondence algorithms. Int. J. Comput. Vision 47 (1/3), 7–42. 10.1023/A:1014573219977

[B39] Scharstein D. Szeliski R. (2003). High-accuracy stereo depth maps using structured light. IEEE Comput. Soc. Conf. Comput. Vision Pattern Recogn. 1, I-195–I-202. 10.1109/CVPR.2003.1211354

[B40] Shibayama M. Watanabe Y. (2007). Estimating the mean leaf inclination angle of wheat canopies using reflected polarized light. Plant Prod. Sci. 10 (3), 329–342. 10.1626/pps.10.329

[B41] Tilneac M. Dolga V. Grigorescu S. Bitea M. A. (2012). 3D stereo vision measurements for weed-crop discrimination. Elektron. Ir Elektrotechn. (Electron. Electr. Eng.) 123 (7), 9–12. 10.5755/j01.eee.123.7.2366

[B42] Vázquez-arellano M. Griepentrog H. W. Reiser D. Paraforos D. S. (2016). 3-D imaging systems for agricultural applications - a review. Sensors 16, 618. 10.3390/s16050618 PMC4883309 27136560

[B43] Wang X. Singh D. Marla S. Morris G. Poland J. (2018). Field - based high - throughput phenotyping of plant height in sorghum using different sensing technologies. Plant Methods, 14 (1), 1–16. 10.1186/s13007-018-0324-5 29997682 PMC6031187

[B44] Weiss M. Baret F. Smith G. J. Jonckheere I. Coppin P. (2004). Review of methods for in situ Leaf Area Index (LAI) determination part II. Estimation of LAI, errors and sampling. Agric. For. Meteorol. 121 (1–2), 37–53. 10.1016/j.agrformet.2003.08.001

[B45] Yanli L. Shaokun L. Jihua W. Jones C. L. Ruizhi X. Zhijie W. (2007). Differentiating wheat varieties with different leaf angle distributions using ndvi and canopy cover. New Z. J. Agric. Res. 50 (5), 1149–1156. 10.1080/00288230709510397

[B46] Yuan W. Li J. Bhatta M. Shi Y. Baenziger P. S. (2018). Wheat height estimation using LiDAR in comparison to ultrasonic sensor and UAS. Sensors 18, 3731. 10.3390/s18113731 PMC6263480 30400154

[B47] Yun L. (2012). Traitement et analyse d'images stéréoscopiques avec les approches du calcul générique sur un processeur graphique (Stereoscopic image processing and analysis with generic computing approaches on a graphical processor). [Thesis]. [Canada]: Ecole Polytechnique de Montreal.

[B48] Zabih R. Woodfill J. (1994). Non-parametric local transforms for computing visual correspondence. in: Computer Vision — ECCV ‘94 151–158. 10.1007/BFb0028345

[B49] Zadoks J. C. Chang T. T. Konzak C. F. (1974). A decimal growth code for the growth stages of cereals. Weed Res. 14, 14, 415–421. 10.1111/j.1365-3180.1974.tb01084.x

[B50] Zhang Z. (2000). A flexible new technique for camera calibration. IEEE Trans. Pattern Anal. Mach. Intell. 22 (11), 1330–1334. 10.1109/34.888718

